# Production of a modified peptide clavanin in *Pichia pastoris*: cloning, expression, purification and in vitro activities

**DOI:** 10.1186/s13568-015-0129-0

**Published:** 2015-08-06

**Authors:** Kelly Cristina Mulder, Loiane Alves de Lima, Priscilla Santos Aguiar, Fábio Correa Carneiro, Octávio Luiz Franco, Simoni Campos Dias, Nádia Skorupa Parachin

**Affiliations:** Centro de Análises Proteômicas e Bioquímicas, Pós-Graduação em Ciências Genômicas e Biotecnologia, Universidade Católica de Brasília, Brasília, DF Brazil; Grupo Engenharia de Biocatalisadores, Departamento de Biologia Celular, Instituto de Ciências Biológicas, Universidade de Brasília, Brasília, DF CEP 70.790-900 Brazil; S-Inova, Pós-Graduação em Biotecnologia, Universidade Católica Dom Bosco, Campo Grande, MS Brazil

**Keywords:** Heterologous expression, Antimicrobial peptide, *Pichia pastoris*, Clavanin

## Abstract

Antimicrobial peptides are one of the most promising peptide-based drugs due to their enormous potential as novel biopharmaceuticals in both human and animal industries. In order to develop strategies to over produce such molecules, heterologous production of a modified version of clavanin A, here named clavanin MO (clavMO), was successfully achieved in the methylothopic yeast *Pichia pastoris*. ClavMO was fused to thioredoxin as a carrier protein and the construction was tested using two promoters, P_AOX1_ and P_GAP_, based on either induced or constitutive expression systems, respectively. After growth in 5 L Bioreactor, clavMO-thio was recovered and purified through size exclusion chromatography. Our findings show that both constitutive and inducible expression systems produce active clavMO fused to thioredoxin against both Gram-negative *Klebsiella pneumoniae* and Gram-positive *Staphylococcus aureus* microorganisms.

**Electronic supplementary material:**

The online version of this article (doi:10.1186/s13568-015-0129-0) contains supplementary material, which is available to authorized users.

## Introduction

Antimicrobial peptides (AMPs) are one of the most promising peptide-based drugs due to their enormous potential as novel biopharmaceutical compounds for the human and animal health industries as well as for their application in agriculture (Agyei and Danquah 2011; Mulder et al. 2013a; Silva et al. 2011a). The increase in interest over these molecules has driven researchers to explore alternatives to chemical synthesis for its large-scale production. The production of AMPs using heterologous systems has many advantages, such as allowing post-translational modification and permitting researchers to develop the best genetic strategy to increase its production, and most importantly, there is an extensive flexibility in microbial systems to be modified and scale-up (Mulder et al. 2013b). The heterologous expression of AMPs has been reported to be successfully performed in diverse organisms such as bacteria, plants and yeast (Parachin et al. 2012).

Among these organisms, the methylotrophic yeast *Pichia pastoris* has been a promising candidate for the heterologous production of AMPs. It has been used for the production of AMPs derived from different sources such as humans (Hong et al. 2007; Kim et al. 2009), mammals (Tang et al. 2012; Zhao and Cao 2012), plants (Cabral et al. 2003; Kant et al. 2009), bacteria (Basanta et al. 2010; Jimenez et al. 2014) and fungi (Varnai et al. 2014; Viragh et al. 2014). From its initial usage in the early 1970s, throughout its complete genome sequence (De Schutter et al. 2009; Mattanovich et al. 2009), to today, *P. pastoris* has become one of the most extensively studied yeasts and presents a versatile system for the production of heterologous proteins (Ahmad et al. 2014). The most common expression vectors use a genetic construction based on either alcohol oxidase I (AOX1) or glyceraldehyde-3-phosphate dehydrogenase (GAP) promoters, named P_AOX1_ and P_GAP_, respectively (Ellis et al. 1985; Waterham et al. 1997). P_AOX1_ is a potent and tightly regulated methanol-inducible promoter; therefore it allows for the controlled expression of foreign proteins, especially when they are toxic to the host. P_GAP_ is constitutive, and one of its reported advantages is that it simplifies cultivation by avoiding the addition of methanol as a carbon source (Zhang et al. 2009). For instance, human cathelicidin (hCAP18) (Hong et al. 2007) and corn defensin (PDC1) (Kant et al. 2009) have been expressed in *P. pastoris* using a constitutive promoter system. On the other hand, many AMPs have been produced using the AOX1 promoter system reaching over 1 g g^−1^ of dry cell weight (DCW), as was recently reviewed (Parachin et al. 2012).

The AMP used in this work was clavanin MO (clavMO) (Silva et al. 2011b), a synthetic variant of amphipathic alpha-helical peptide clavanin A (clavA). ClavA was first isolated from hemocytes of the tunicate *Styela clava*. The peptide clavA presents, among its 23 residues, strategically placed histidine residues which provide its pH-dependent antimicrobial activity, as well as glycine and phenylalanine residues which confer to this peptide a relative conformational flexibility and hydrophobicity, thus facilitating its insertion into the target membrane (van Kan et al. 2003b). ClavMO is 5 amino acid residues longer than clavA, and it has been shown to have higher antibacterial activity against both Gram-negative *Klebsiella pneumoniae* and Gram-positive *Staphylococcus aureus*. Furthermore, clavMO has also presented immunomodulatory, antitumor and antiviral activities (Silva et al. 2011b).

Due to its high potential as a new antimicrobial peptide, a large amount of clavMO is currently required for the production of different drug-delivery systems. Therefore, the aim of this study was to establish a system for large-scale production of heterologous clavMO in *P. pastoris*. For that, two promoters, P_AOX1_ and P_GAP_ were tested based on two expression systems, induced and constitutive, respectively. ClavMO was fused to thioredoxin as a carrier protein, chosen for its properties of increasing both stability and solubility of heterologous proteins (Esposito and Chatterjee 2006). Our findings show that the induced system produces active clavMO against the microorganisms *K. pneumoniae* and *S. aureus*.

## Materials and methods

### Strains and plasmids

Bacterial strains and plasmids used in this work are presented in Table 1. The strains were grown at 37°C in Luria broth medium (0.5% Yeast extract, 1% Peptone and 1% Sodium Chloride), and the yeast strains were grown at 28°C in either YPD (0.5% Yeast extract, 1% Peptone and 2% Dextrose) or BMGY media (2% Peptone, 1% Yeast extract, 100 mM Potassium phosphate pH 6, 1.34% Yeast Nitrogen Base (w/o AA), 0.4 µg/mL Biotin, 1% Glycerol). When necessary, the media were supplemented with the appropriate antibiotics (ampicillin for *Escherichia coli* cultivations at 100 µg/mL, and zeocin for *P. pastoris* cultivation at 100 µg/mL). The gene containing the carrier protein thioredoxin fused to the peptide clavMO was cloned into both expression vectors pPICZαA and pGAPZαB, under the methanol-inducible P_AOX1_ promoter and the constitutive P_GAP_ promoter, respectively.

**Table 1:** Strain and plasmids used in this work

Strain and plasmids	Genotype	Reference
Strains		
*E coli*		
XL1-Blue	EndA1 gyrA96(nal^R^) thi-1 recA1 relA1 lac glnV44 F’[::Tn10 proAB^+^ lacI^q^ Δ(lacZ)M15] hsdR17(r_K_^−^ m_K_^+^)	Stratagene
*P. pastoris*		
X33	Wild type	
X33/pPICZαA-clavMO	X33 transformed with the plasmid pPICZαA-clavMO	This work
X33/pGAPZαB-clavMO	X33 transformed with the plasmid pGAPZαB-clavMO	This work
Plasmids		
pPICZα A	Fator-α as secretion signal, AOX1 promotor, ble^r^	
pGAPZα B	Fator-α as secretion signal, P_GAP_ promotor, ble^r^	
pPICZαA-clavMO	pPICZαA with the gene coding for the cassette thioredoxin-clavanin MO	This work
pGAPZαB-clavMO	pGAPZαB with the gene coding for the cassette thioredoxin-clavanin MO	This work

### Cloning

**Figure 1:**
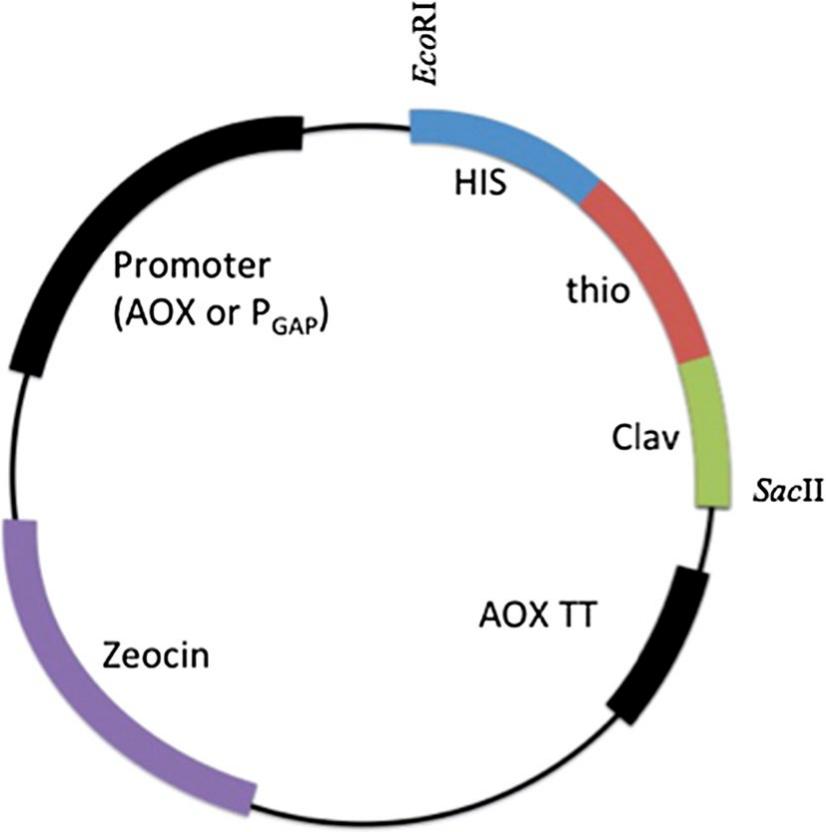
Plasmid vector map. The constitutive system was developed using the plasmid pGAPZαB, which contains the P_GAP_ promoter. The inducible system was constructed using the pPICZαA, which contains the P_AOX_ promoter. *HIS* his-tag, *Trx* thioredoxin *E. coli* gene, *Cla* clavanin gene, *AOX TT* terminator.

The thio-clavMO gene sequence was synthesized by Epoch Life Science (Additional file 1: Figure S1). To clone the gene into both plasmids pPICZαA and pGAPZαB, the restriction sites *Eco*RI and *Sac*II were used, resulting in the expression vectors pPICZαA-clavMO and pGAPZαB-clavMO (Figure 1; Table 1). Both plasmids were firstly transformed into *E. coli* XL1-Blue strains and sequenced to confirm the insertion of the expression cassette.

### Yeast transformation

The vectors whose constructions were confirmed by sequencing were inserted into the *P. pastoris* X-33 strain using the electroporation method as described by Invitrogen™. Briefly, cells of the X-33 strain were grown in solid YPD at 28°C for 2–3 days. A single colony was then grown in 5 mL of YPD overnight at 30°C at 250 rpm. 0.1 to 0.5 mL of this culture was inoculated into 100 mL of YPD and grown at 30°C at 250 rpm until an OD_600_ of 1.3–1.5 was reached. The cells were centrifuged at 1,500*g* for 5 min and washed three times with ice-cold distilled water and then resuspended in 1 M cold sorbitol. Plasmids to be inserted in *P. pastoris* were linearized with *Avr*II and *Bgl*II for pGAP and pPIC, respectively. About 10 μg of linearized DNA was added to 80 μL of competent cells and 320 μL of 1.0 M sorbitol. Electrical shock using 0.2 cm cuvette was performed using Gene Pulser II (Biorad). After electric shock, 1 mL of 1 M cold sorbitol was added to the cells followed by incubation at 30°C. After 1 h of incubation, cells were plated in solid YPD medium supplemented with zeocin 100 µg/mL and stored at 28°C for 2–3 days.

### Evaluation of clavMO production

In order to evaluate the production of clavMO in both plasmids, a single colony from each X33/pPICZαA-clavMO and X33/pGAPZαB-clavMO (Table 1) was inoculated into YPD medium at 28°C and cultivated overnight at 200 rpm of agitation. Both strains were inoculated in 100 mL BMGY to reach an OD_600_ of 0.2 and grew for 96 h. Samples were collected at times 0, 24, 48, 70 h. The pPICZαA-clavMO strain was induced every 24 h with methanol 100% to a final concentration of 0.5% in order to induce the gene encoding for clavMO cloned under the AOX1 promoter.

### Protein quantification, SDS-PAGE and immunoblot assays

Protein quantification was performed by using Qubit-Invitrogen™ according to manufacturer’s instructions. For the Western blot and SDS-PAGE experiments, 150 µg/mL of protein from cultured supernatant was collected by the TCA (trichloroacetic acid) precipitation method using TCA at 75%. The pellet was washed twice with ice-cold acetone and dissolved in 25 μL 3× Laemmli buffer (Laemmli 1970), boiled for 10 min, centrifuged briefly and loaded onto an SDS-PAGE gel using protein molecular weight (Thermo Scientific™). SDS-PAGE was silver stained as previously described (Blum et al. 1987). In order to detect proteins, the protein samples were first separated by SDS-PAGE and then transferred under semi-dry conditions onto a nitrocellulose membrane using electroblotting (Towbin et al. 1979). The electrophoretic transfer of proteins was performed in the Trans-Blot SD (Biorad) and carried out for 15 min at 13 V, 3 A and 300 mA using Blotting buffer (3 g Tris, 14 g glycine, 20% (v/v) methanol, 0.1% (w/v) SDS). After transference, membrane blocking was achieved by incubation in AP-T buffer (1 M Tris/HCl pH 7.4, 1 M NaCl, 25 mM MgCl_2_, 0.03% (v/v) Tween 20) containing 5% (w/v) milk powder. The following steps were then carried out only in AP-T buffer. The procedure for detection of labeled proteins was performed using polyclonal antibodies against thioredoxin (1:5,000) and revealed using BCIP/NBT solution, according to the manufacturer’s protocol.

### Growth of X-33 pPicZ-clavMO in 5 L Bioreactor

The strain X-33/pPic-clavMO was grown in a 5 L bioreactor (Bio Flo115, New Brunswick). For cultivation in the bioreactor, BMGY media was used containing 40 g l^−1^ glycerol. Pre-growth was performed in 100 mL BMGY shake flasks for 30 h. The cells were centrifuged and inoculated in the bioreactor to reach an initial OD_600_ of 0.5. Glycerol was used for 24 h of growth, after which methanol was fed into the bioreactor for a final concentration of 0.5% every 12 h up to 72 h. At the end of the fermentation, 5 L culture medium was centrifuged at 10,000 rpm for 10 min. The supernatant was concentrated 10-fold using Quickstand (GE Healthcare Life Sciences) by diafiltration using hollow fiber cartridge with a cut-off of 3,000 NMWC.

### Peptide isolation

Concentrated supernatant from the culture of the strain X-33/pPic-clavMO containing 5 mg/mL of protein extract was lyophilized and injected into a size exclusion chromatography Äkta purifier (GE Healthcare). The column utilized was the Hiload™ 16/60 Superdex™ 75 prep grade column (GE Healthcare). This was equilibrated with filtered and degassed water MilliQ at room temperature. The flow rate utilized was 0.8 mL/min. Fractions were eluted with 5 mL volumes, totaling 22 fractions. These were monitored at 216 and 280 nm for 270 min. The fractions from 16 to 22 were lyophilized and used for further antibacterial assays.

### Antibacterial assays

The antibacterial assays were carried out by microdilution assay using an Elisa reader for 96-well microplates (Biotek, USA). Microdilution assays were performed according to the standards of the CLSI (Clinical Laboratory Standards Institute) (2010; 2012). Antimicrobial activity assays were performed using *K. pneumoniae* (ATCC13883) and *S. aureus* (ATCC25923). For each assay, chloramphenicol (30 µg/mL) was used as positive control (C+) and MH broth at pH 7.3 as a negative control. Bacterial growth was monitored every 30 min until it reached the stationary phase (about 24 h). The protein fractions 16–22 were utilized in a concentration of 120 µg/mL. Percentage of bacterial growth inhibition values was based on the absorbency values at 625 nm, which were compared with the values obtained for C+ (representing 100% bacterial growth inhibition). All antibacterial assays were performed in triplicate.

## Results

### Production of clavMO

**Figure 2:**
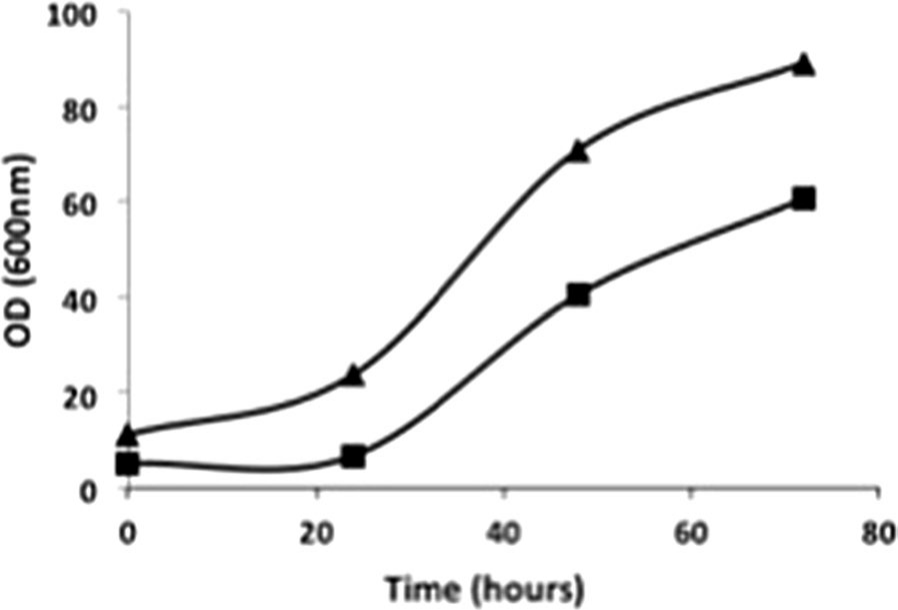
Growth of *P. pastoris* strains X33/pPICZαA-clavMO (P_AOX_, *triangle*) and X33/pGAPZαB-clavMO (P_GAP_, *square*). Growth was monitored by OD_600_ every 24 h. Methanol (0.5%) was added to *P. pastoris* strain X33/pPICZαA-clavMO after 30 h of growth. Experiments were done in triplicate where figure shows the growth profile within 10% standard deviation.

X-33/pPICZαA-clavMO and X-33/pGAPZαB-clavMO were selected for growth experiments and heterologous peptide production. *P. pastoris* containing the constitutive expression cassette, pGAPZαB-clavMO, resulted in about 1.5-fold lower final OD when compared to the strain containing the inducible expression cassette pPICZαA-clavMO (Figure 2). Therefore for the evaluation of antibacterial activity of clavMO the strain pPICZαA-clavMO was chosen.

### Analysis of recombinant clavMO by Western blot

**Figure 3:**
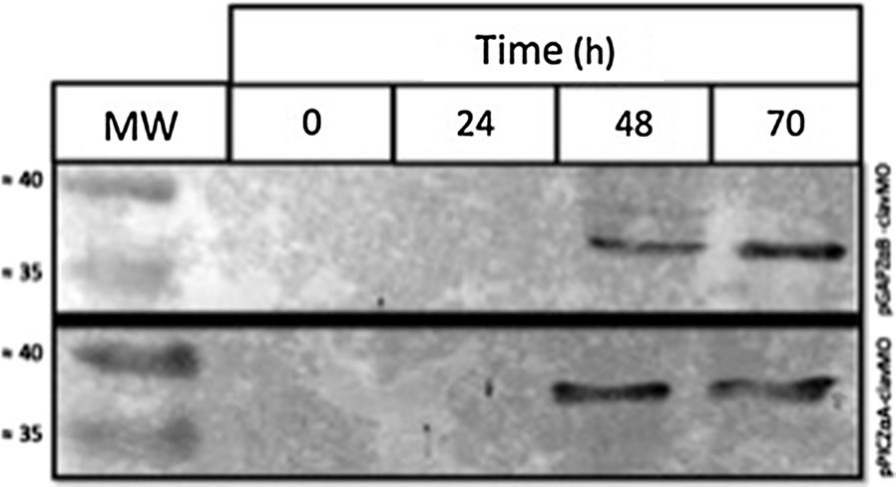
Western blot assay of the supernatant of the strains X33/pGAPZαB-clavMO (*upper panel*) X33/pPICZαA-clavMO (*lower panel*). Samples were collected at times 0, 24, 48 and 70 h. *MW* molecular weight (kD). Antibody anti-his tag 1:5,000

Supernatant of the strains X33/pPICZαA-clavMO and X33/pGAPZαB-clavMO were collected at 0, 24, 48 and 70 h. Samples of 150 µg/mL from supernatant from the culture were applied into an SDS-PAGE (12%) prior Western blot assay. According to the results obtained, it can be observed that the expression of the recombinant clavMO was detected at 48 and 70 h in both strains while no recombinant protein was detected prior induction with Methanol (Figure 3). It was also observed that the appropriated time for induction of the promoter AOX1 in the X33/pPICZαA-clavMO strain was after 30 h when all initial glycerol was entirely consumed (Figure 3, lower panel), as well as the constitutive expression of the promoter P_GAP_ for production of the recombinant clavMO in the X33/pGAPZαB-clavMO strain (Figure 3, upper panel). The predicted size of Thioredoxin-ClavMO fused gene was 34.47717 kDa (http://web.expasy.org/compute_pi/). Our Western blot results show that the detected protein correspondent to our expression cassette was between 35 and 40 kda (Figure 3), therefore, bigger than the predicted MW.

### Purification

**Figure 4:**
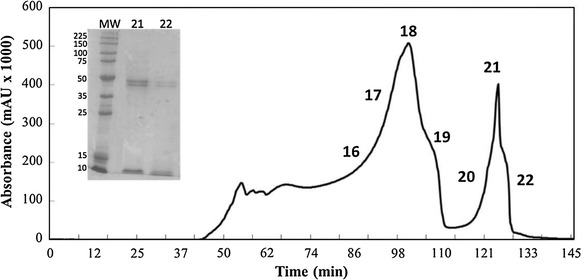
Chromatogram profile of supernatant of strain X-33/pPic-clavMO. 5 mg/mL of protein extract using a Hiload™ 16/60 Superdex™ 75 prep grade column (GE Healthcare) originating 24 fractions eluted in approximately 5 mL and monitored at 280 nm. The silver-stained gel shows the protein recovered from fractions 21 and 22 after purification process. *MW* molecular weight (kD).

Figure 4 shows the profile of the *P. pastoris* supernatant containing X33/pPICZαA-clavMO after 70 h of cultivation. From purification of 5 mg/mL of protein extract into a size exclusion chromatography, seven fractions were collected, numbered 16–22, and were selected for further analysis. After lyophilization, these samples were used to perform antibacterial assays, and two fractions, 21 and 22, were shown to have antibacterial activity. SDS-PAGE analyses of theses samples had shown the protein band corresponding to the molecular mass of clavMO but with different purity degrees (Figure 4).

### Antibacterial assays

Preliminary bioassays revealed that some fractions showed low protein quantification and no antibacterial activity. Therefore, the fractions ranging from 16 to 22 were chosen for antibacterial bioassay due to their higher absorbance at 280 nm. Bioassays were performed against Gram-negative *K. pneumoniae* and Gram-positive *S. aureus* bacteria. For each bioassay, 120 µg/mL of X-33/pPic-clavMO protein was used in triplicate. The bioassay against *K. pneumoniae* showed antibacterial activity for fraction 21 and 22 where bacterial growth was reduced by 56 and 8%, respectively (Table 2). On the other hand, the bioassay performed against *S. aureus* showed antibacterial activity for fractions 21 and 22 where bacterial growth was inhibited by 68 and 89%, respectively (Table 2).

**Table 2:** Antibacterial assay against *K. pneumoniae* and *S. aureus* using 120 µg/mL of X-33/pPic-clavMO protein for 24 h at absorbance λ = 625 nm

Antibacterial activity (%)		
Fractions X-33/pPic-clavMO	*Klebsiella pneumoniae* (ATCC13883)	*Staphylococcus aureus* (ATCC25923)
16	NS	NS
17	NS	NS
18	NS	NS
19	NS	NS
20	NS	NS
21	56 ± 1.4	68 ± 1.7
22	8 ± 1.2	89 ± 1.4

Values were represented by mean ± standard deviation. Chloramphenicol (30 µg/mL) and MH broth, pH: 7.3, as positive and negative controls, respectively.

*NS* not significant.

## Discussion

Heterologous production of antimicrobial peptides has been attempted in several hosts over the last few years. After the bacterium *Escherichia coli*, yeasts are the second most used system for heterologous peptide production (Parachin et al. 2012). In this study, heterologous production of a modified clavanin fused to thioredoxin, clavMO-thio, was initially attempted in different *E. coli* strains. Nevertheless, expression of the encoding gene could never be confirmed (Additional file 2: Figure S2, Additional file 3: Figure S3 and Additional file 4: Figure S4). The same has been previously observed when the production of SPE10 isolated from the *Pachyrrhizus erosus* peptide was attempted in both *E. coli* and *P. pastoris* where heterologous peptide production could only be confirmed when produced in yeast (Song et al. 2005).

Although *Sacharomyces cerevisiae* is the most common yeast utilized for biopharmaceutical production, the yeast *P. pastoris* was chosen as the host in this study for having a GRAS (Generally Regards As Safe) status, the ability to grow in high cell density cultures (since it does not present fermentative behavior), and its reported high levels of secreted recombinant protein, which simplifies downstream purification processes (Ahmad et al. 2014). Finally, most recombinant AMPs produced in yeast use *P. pastoris* as a host (Parachin et al. 2012).

Although some companies claim the production of recombinant clavanins isoforms such as B, C, D and E using both *S. cerevisiae* and *E. coli* as hosts, herein we report for the first time the heterologous production of clavanin using *P. pastoris* as a host to express clavMO. Synthetic ClavMO is reported to have higher antibacterial activity against both Gram-positive (e.g., 78.75 µM against *S. aureus* ATCC29213; 2.5-fold higher than synthetic clavA) and Gram-negative bacteria (e.g. 39.40 µM against *K. pneumoniae*—ATCC13885; 2.5-fold higher than synthetic clavA). Furthermore, clavMO has also presented immunomodulatory, antitumor, antiviral and insecticide activities (Silva et al. 2011b).

In this study Clav-MO fused to thioredoxin in its N-terminal presented antibacterial activity. Although one could argue that thioredoxin could have inhibitory activity by itself its gene sequence was from the *E. coli* genome where it has been previously described its role in defense against oxidative stress or in control of apoptosis (Arnér and Holgren 2000). Moreover thioredoxin is frequently used as a carrier protein for production of recombinant antimicrobial peptide representing more than 20% of all reported fusion expressions of antimicrobial peptides (Li 2009). Finally, in a study for heterologous production of viscotoxin where 13 fusion proteins were tested, thioredoxin gave the highest yield of soluble protein (Bogomolovas et al. 2009). For all those reasons we claimed that the activity against the microorganisms tested in our work is derived from the activity of the peptide clav-MO.

Here we demonstrate the production of a heterologous AMP using both constitutive and inducible promoters. Few of the reported studies used a constitutive promoter to express gene encoding for AMPs (Guo et al. 2012; Hong et al. 2007; Yu et al. 2010). Constitutive AMP production is advantageous as the fermentation process is facilitated because there is no need for additional inductor or media exchange. Nevertheless, if the AMP has antifungal activity, the use of such a promoter is not advisable, for it impairs yeast growth and consequently heterologous AMP production. In all reported cases, the amount of heterologous peptide produced using constitutive promoter was not reported. Moreover, in this study the strain with constitutive cassette had a negative impact on final OD, which corroborates previous studies and reinforces the utilization of inducible constructions for heterologous production of AMP.

Regarding clavanin mode of action, it is known that its bactericide activity is related to membrane stability in a pH dependent form (van Kan et al. 2001, 2002, 2003a). Furthermore, clavanin A has been shown to interact with lipid bi-layers, resulting in drastic changes in membrane morphology (van Kan et al. 2003b). Recently, our group has shown that nanoformulated clavanin A inhibit bacterial growth of *S. aureus, K. pneumoniae* and *Pseudonomas aeruginosa*, being an excellent candidate for treating patients contaminated with antibiotic-resistant bacteria (Saúde et al. 2014). Another work form our group has shown that clavanin A is effective in treatments of wound and sepsis infections by avoiding the beginning of sepsis, and as consequence, it reduces mortality (Silva et al. 2015).

Nevertheless, the chemical synthesis of clavanin aiming at nanoformulation is not cost-effective. In this study, recombinant clavMO was shown to inhibit both Gram-positive and Gram-negative bacteria. Thus, it presents an initial step for the development of cost-effective, large-scale production of this AMP.

In this study, for the first time, the modified version of clavanin A, clavMO was heterologously produced in *P. pastoris* using both constitutive and inducible expression cassettes. Both systems yielded protein detection in yeast supernatant by Western blot assays. The strain with integrated constitutive construction resulted in lower final OD when compared to the strain with the inducible construction integrated in its genome. Therefore, the strain producing clavMO after induction with metanol was chosen for the following experiments. ClavMO was produced in a 5 L scale followed up by purification using gel filtration. Finally, antimicrobial assays showed that recombinant clavMO could inhibit up to 56 and 89% Gram-negative and Gram-positive bacteria, respectively. Conclusively, it is evident that *P. pastoris* is an excellent host for the functional production of clavMO, and that this system may be utilized for further scale-up production of AMPs.

## Additional files

Additional file 1:
**Figure S1.** DNA nucleotide sequence for thioredoxin and clavMo (underlined). Start codons of both carrier and peptide sequence are indicated in bold. Stop codon is highlighted in red Additional file 2:
**Figure S2.** A) Expression vector pET21a(+) used as backbone to insert the expression cassette; B) Expression cassette comprised of a signal sequence (Ss), a HIS-tag, the thioredoxin gene end the ClavMO peptide. In the white boxes are presented the restriction sites Additional file 3:
**Figure S3.** Western blot analysis of the supernatant fraction of lysed *E. coli* strains. A) pLyzS/pET21-clavMO, B) pRIL/pET21-clavMO Additional file 4:
**Figure S4.** Western blot analysis of the pellet cell fraction of lysed *E. coli* strains. A) pLyzS/pET21-clavMO, B) pRIL/pET21-clavMO

## Abbreviations

AMP: antimicrobial peptide
ClavMO: clavanin modified
ClavA: clavanina A
AOX1: alcohol oxidase I
GAP: glyceraldehyde-3-phosphate dehydrogenase
