# Supplementary material for: Production of a modified peptide clavanin in Pichia pastoris: cloning, expression, purification and in vitro activities
Source: AMB Express. 2015 Aug 6;5:46. doi: 10.1186/s13568-015-0129-0 (PMC4524883; doi:10.1186/s13568-015-0129-0)
Supplement: Additional file 3: — Figure S3. Western blot analysis of the supernatant fraction of lysed E. coli strains. A) pLyzS/pET21-clavMO, B) pRIL/pET21-clavMO [file 13568_2015_129_MOESM3_ESM.docx]

**AMB Express**

**Additional file**

**Production of a modified peptide clavanin in *Pichia pastoris*: cloning, expression, purification and in vitro activities**

Kelly Cristina Mulder^1^, Loiane Alves de Lima^1^, Priscilla Santos Aguiar^2^, Fábio Correa Carneiro^2^, Octávio Luiz Franco^1,3^, Simoni Campos Dias^1^ and Nádia Skorupa Parachin^1,2*^.

^1^Centro de Análises Proteômicas e Bioquímicas, Pós-Graduação em Ciências Genômicas e Biotecnologia, Universidade Católica de Brasília, Brasília-DF, Brazil.

^2^ Grupo Engenharia de Biocatalisadores, Departamento de Biologia Celular, Instituto de Ciências Biológicas, Universidade de Brasília, Brasília, Brazil.

^3^S-Inova, Pós-Graduação em Biotecnologia, Universidade Católica Dom Bosco, Campo Grande, MS, Brazil

^*^Corresponding author: Grupo Engenharia de Biocatalisadores, Departamento de Biologia Celular, Instituto de Ciências Biológicas, Universidade de Brasília, CEP 70.790-900 Brasília-DF, Brazil. Phone: +55(61)3448-7126. E-mail: [nadiasp@unb.com.br](mailto:nadiasp@unb.com.br); [nadiasp@gmail.com](mailto:nadiasp@gmail.com)

**Supplementary Figures 1 to 4 (S1 – S4)**


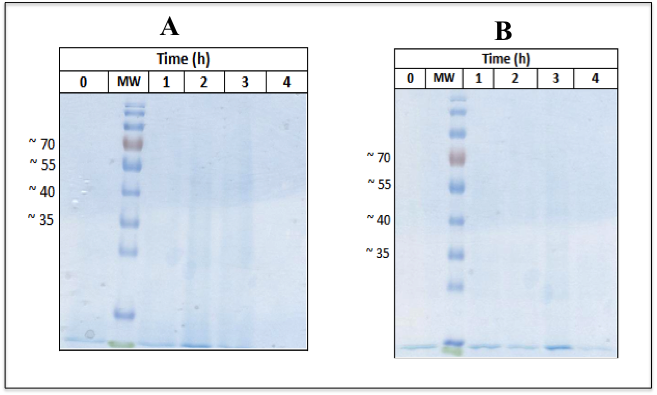


**Figure S3** Western blot analysis of the supernatant fraction of lysed *E. coli* strains. A) pLyzS/pET21-clavMO, B) pRIL/pET21-clavMO
